# Association between elevated blood glucose level and brain injuries (IVH/WMI) in preterm infants

**DOI:** 10.1186/s12887-026-07341-0

**Published:** 2026-07-16

**Authors:** Renée Lampe, Ursula Felderhoff-Müser, Irina Sidorenko, Marcus Krüger, Eva Lück, Ekaterini Michales, Uwe Sassen, Christian Brickmann

**Affiliations:** 1 TUM School of Medicine and Health, Department of Orthopedics and Sport Orthopedics, Markus Würth Professorship at Technical University of Munich, Research Unit for Paediatric Neuroorthopedics and Cerebral Palsy of the Buhl-Strohmaier Foundation, Technical University of Munich, TUM University Hospital, Ismaninger Str. 22, Munich, 81675 Germany; 2 Department of Paediatrics I, Neonatology, Paediatric Intensive Care, Paediatric Infectious Diseases, Paediatric Neurology, Centre for Translational Neuro- and Behavioural Sciences, University of Duisburg-Essen, University Hospital Essen, Hufelandstrasse 55, 45122 Essen, Germany; 3 TUM School of Medicine and Health, Department of Orthopedics and Sport Orthopedics, Research Unit for Paediatric Neuroorthopedics and Cerebral Palsy of the Buhl-Strohmaier Foundation, Technical University of Munich, TUM University Hospital, Ismaninger Str. 22, 81675 Munich, Germany; 4 Clinic for Neonatology, Munich Clinic Harlaching & Schwabing, Sanatoriumsplatz 2, 81545 Munich, Germany; 5 TUM School of Medicine and Health, Department of Neonatology, Technical University of Munich, Ismaninger Str. 22, 81675 Munich, Germany; 6 TUM School of Medicine and Health, Department of Pediatrics, Kinderklinik Muenchen Schwabing, Technical University of Munich, TUM University Hospital, Munich, Germany

**Keywords:** Extremely and very preterm infants, Intraventricular hemorrhage, White matter injury, Whole blood glucose, Hypoglycemia, Hyperglycemia

## Abstract

**Introduction:**

The likelihood of developing preterm brain injury such as intraventricular hemorrhage (IVH) or cystic white matter injury (cWMI) largely depends on gestational age. However, its pathogenesis is multifactorial, and accounting of additional risk factors is important for preventing brain injury. Glucose is one of the most important energy sources for the brain. Both high and low blood glucose level may be associated with the onset and progression of brain disorders.

**Methods:**

To evaluate the relation between preterm brain injury (IVH/cWMI) and blood glucose level, univariate statistical analyses of retrospective data of 109 extremely and very preterm infants was performed. The analyzed data included 14 prenatal and infant diagnoses and 32 regularly measured parameters.

**Results:**

Mean values of whole blood glucose levels in preterm infants diagnosed with IVH or cWMI was higher than in controls indicating that elevated blood glucose level is associated with development of preterm brain injury. Mean values were higher both before and after diagnosis of IVH or cWMI, and even significantly exceeded the hyperglycemia threshold of 125 mg/dL following IVH diagnosis. Furthermore, a significant or moderate association was revealed between elevated blood glucose level and 8 medical diagnoses, as well as 21 routinely measured parameters.

**Conclusion:**

Our study emphasizes the importance of regularly monitoring blood glucose levels in preterm infants and keeping it within a safe range.

## Introduction

Glucose is essential for fetal growth and development and represents one of the most important energy sources for the brain [1]. Newborns have a particularly high glucose requirement owing to their large brain volume relative to body weight [2]. Unlike term newborns, who adapt to interrupted transplacental glucose transfer through hormonal and metabolic processes, preterm infants struggle to maintain glucose homeostasis due to markedly limited glycogen stores, which ordinarily accumulate only during the third trimester [1].

Beyond typical physiological instability, pathological and medication-induced glucose fluctuations carry serious consequences for neurodevelopment. Hyperglycemia may result from stressors such as sepsis, cerebral hemorrhage, excessive glucose administration, or medications such as corticosteroids and catecholamines, and is associated with white matter injury (WMI) [3]. Immaturity of insulin regulation encompasses both insufficient insulin supply and immature insulin receptors and means that hyperglycemia can arise in the absence of other disease and may contribute to the development of cystic WMI (cWMI) [4]. Even mild disturbances to glucose homeostasis may impair neuronal metabolism and cause structural brain damage [5].

The reported incidence of neonatal hypo- and hyperglycemia varies widely. Hyperglycemia affects up to 80% of extremely preterm infants and is associated with increased mortality and reduced brain white matter volume at term [6, 7]. Estimates for hypoglycemia range from 17% to 41%, and for hyperglycemia from 20% to 86% [7, 8]. In this context, fluctuations in blood sugar levels can be assessed more precisely using such measures as mean amplitude of glycemic excursions (MAGE). MAGE is a widely used measure of glycemic variability, reflecting the average amplitude of glucose excursions, and is most appropriate for analyses based on closely spaced or continuous glucose measurements. For example, Musso et al. [9] applied this MAGE approach to characterize glycemic variability in preterm infants receiving continuous glucose monitoring. Although MAGE provides a valuable conceptual context, this parameter was not analysed in the present study.

Serum-glucose fluctuations are associated with increased morbidity and mortality, as well as impaired neurological development, potentially leading to brain damage, cerebral palsy, developmental delay, neurobehavioural and neuropsychiatric deficits [10, 11]. The study of Burns et al. [12] have demonstrated white matter abnormalities and cerebral hemorrhages in symptomatic hypoglycemic newborns as early as nine days postpartum.

Several studies have examined the relationship between glucose instability and intraventricular hemorrhage (IVH). Al-Mouqdad et al. [13] found that among 554 very preterm infants, 28% developed IVH, with severe IVH significantly associated with hyperglycemia. Zhu et al. [14] reported hyperglycemia in 44.7% of preterm infants, with a significantly higher IVH incidence compared to normoglycemic infants. Logistic regression identified severe hyperglycemia (≥ 220 mg/dL) as an independent risk factor for IVH, with both duration and severity influencing risk [11, 13].

Pathophysiologically, hypoglycemia induces cerebral vasodilation and hyperperfusion as a compensatory response to cerebral energy crisis, potentially precipitating IVH [15]. Hyperglycemia, conversely, fosters a pro-inflammatory vascular environment in which hemodynamic fluctuations more readily cause microvessel rupture [16]. Defining critical glucose thresholds remains contested. Hypoglycemia in neonates is generally indicated by plasma glucose below 2.6 mmol/L (47 mg/dL), though definitions vary across studies [17, 18]. Neonatal hyperglycemia is diagnosed when whole blood level exceeds 6.9 mmol/L (125 mg/dL) [19] or plasma glucose level exceeds 8.3 mmol/L (150 mg/dL). Hyperglycemia occurs most frequently in infants born before 28 weeks of gestation (WG) during the first two weeks of life [16].

In this study we adhere to these hypo- and hyperglycemia thresholds published in scientific literature, but not to the intervention thresholds used in clinical practice. Our goal is to analyze the stress and risk of abnormal glucose levels in preterm infants with regard to IVH and cWMI. The necessity or effect of therapy, as well as the method of glucose administration, are not part of our investigation. This deliberate distinction is supported by the study by Nakajima et al. [20], which found no significant association between insulin therapy for hyperglycemia and mortality or neurological impairment in extremely preterm infants. This suggests that therapeutic decisions do not directly allow conclusions to be drawn about the underlying pathophysiological relevance of specific glucose levels.

The aim of this study is to investigate the relationship between blood glucose levels and IVH/cWMI in preterm infants. Our primary focus is to determine whether hypo- or hyperglycemia represent significant risk factors for these brain injuries, independent of therapeutic interventions and nutritional management. Accordingly, the focus of our work is on the relationship between spontaneous glucose deviations and the risk of brain damage, independent of therapeutic interventions and dietary habits.

### Materials and methods

This study presents a retrospective analysis of blood glucose levels documented during a predefined early postnatal observation period in preterm infants treated at two Level III perinatology centers in Germany. Blood glucose values were extracted from routine clinical records and reflected standard monitoring during neonatal care. The number and timing of these measurements therefore varied according to the infant’s clinical status, postnatal age and gestational age at birth. Rather than evaluating treatment effects or therapeutic strategies, we examined whether abnormal glucose levels were associated with preterm brain injury, specifically IVH or cWMI.

Clinical records during first two weeks of life were collected from 109 preterm infants born between 22 + 4 and 34 + 4 WG with birth weight between 490 g and 1950 g. IVH (grades I-IV according to Papile classification [21]) and cWMI were diagnosed by cranial ultrasound, routinely performed on day 1st, 3rd, 7th, 14th and 28th of life. Our cohort did not include magnetic resonance imaging (MRI) data, and the classification of IVH and cWMI relied exclusively on cranial ultrasound. Additional examinations were also carried out depending on the clinical status. The present research aims to analyze the relationship between glucose levels and the presence of IVH/cWMI. Therefore, the study cohort is composed with the high number of IVH/cWMI cases which does not reflect the frequency of IVH or cWMI in an unselected group of preterm infants.

Infants without IVH/cWMI diagnoses are referred here as control group (*n* = 43). In the study cohort we have 38 patients with IVH (grades I-II), 18 patients with IVH (grades III-IV), 25 patients with cWMI, and 66 patients with at least one of these diagnoses. We compared the control group with each of three patient groups, designated as the IVH group, the cWMI group, and the combined IVH/cWMI group, respectively. For each patient group, whole blood glucose levels were compared over a two-week period, and separately for the days preceding and following ultrasound diagnosis.

Clinical data and regularly measured parameters were retrospectively extracted from patient charts. Clinical diagnoses (such as acidosis, sepsis, disseminated intravascular coagulation, and thrombocytopenia) and regularly measured parameters were recorded as documented in the medical records according to standard clinical practice. Three parameters, cerebral blood flow (CBF), its fluctuations (CBF-CBF_mean_) and partial pressure in brain tissue (PtO_2_) were calculated using previously developed mathematical models [22].

Blood glucose measurements were taken from clinical patient records as continuous numerical variables, rather than as data grouped into predefined diagnostic categories. For descriptive purposes and clinical interpretation, we consider these continuous values in relation to published neonatal reference safety ranges (40–125 mg/dL) [19, 23]. In our study, we further categorize a condition in which the whole blood glucose level exceeds 125 mg/dL as hyperglycemia, and a condition in which it is below 40 mg/dL as hypoglycemia.

All data records were thoroughly verified using clinically established plausible ranges, and typing mistakes and outliers were filtered out. Continuous parameters are presented as mean value, standard deviation, median and interquartile range (IQR=Q_3_-Q_1_, where lower quartile Q_1_, median Q_2_ median and upper quartile Q_3_ are 25th, 50th and 75th percentiles, respectively). Clinical diagnoses are presented as the number of cases and percentages.

In our dataset, blood glucose levels were not recorded continuously, but only several times a day. Depending on the patient’s condition, the number of measurements per patient varied from one to seven per day. To address these differences in clinical records, we performed patient-level averaging of all parameters over all days, thus aggregating the data into a single summary statistic per infant for the observation period. This shift from treating individual measurements as independent points to using patient-level averages is a necessary step toward mitigating pseudoreplication.

To determine the association between whole blood glucose levels and brain injury (IVH/cWMI), a univariate analysis is performed using the two-sided Wilcoxon rank sum test with a significance level of 5%, which corresponds to a p-value of 0.05. All statistical analysis was performed using official MATLAB25b software.

## Results

Descriptive statistics of diagnoses and regularly measured parameters of the entire cohort of 109 patients are summarized in Tables 1 and 2, respectively. Controls were matched for gestational age and birth weight. Therefore, the differences between the mean values of these parameters in the control group and each of the affected groups are not statistically significant (Table 3). Whole blood glucose levels for controls and affected infants, their association with regularly measured parameters and diagnoses are presented in Tables 3, 4 and 5 and compared with threshold values of hypo- and hyperglycemia from the literature (Table 6).

**Table 1:** Descriptive statistics of the entire study cohort

Parameters and diagnosis	Patient cohort *n* = 109 (100%)		
	min	max	mean ± std
*Prenatal or birth parameter*			
Gestational age [weeks]	22 + 4	33 + 4	26.9 ± 2.6
Birth body weight [g]	490	1950	966 ± 319
APGAR 1 min	1	9	5.0 ± 2.1
APGAR 5 min	1	10	6.9 ± 1.7
APGAR 10 min	3	10	8.1 ± 1.2
Umbilical cord pH	6	7.47	7.3 ± 0.2
Blood glucose [mg/dL]	25.7	678	123.2 ± 56.1
Male sex	65 (59.63%)		
Multiple pregnancy	40 (36.69%)		
Sectio	96 (88.07%)		
Surfactant administration	96 (88.07%)		
*Prenatal diagnosis*			
Chorioamnionitis/amniotic infection syndrome	76 (69.72%)		
Preterm premature rupture of membranes (PPROM)	59 (54.13%)		
In vitro fertilisation	14 (12.84%)		
Preeclampsia/EPH-gestosis	11 (10.09%)		
Intrauterine growth restriction (IUGR)	6 (5.51%)		
*Infant diagnosis*			
IVH grades I-IV	56 (51.38%)		
IVH grades I-II	38 (34.86%)		
IVH grades III-IV	18 (16.51%)		
cWMI	25 (22.94%)		
IVH (I-IV) and cWMI	15 (13.76%)		
IVH (I-IV) or cWMI	66 (60.55%)		
Respiratory distress syndrome (all grades)	94 (86.24%)		
Erythrocyte transfusion requirement	67 (61.46%)		
Sepsis	36 (33.03%)		
Bronchopulmonary dysplasia (BPD, all grades)	29 (26.61%)		
Respiratory Adaptation Disorder	29 (26.61%)		
Acidosis (respiratory, metabolic, or both)	23 (21.10%)		
Necrotizing enterocolitis (NEC)	19 (17.43%)		
Pulmonary hemorrhage	13 (11.93%)		
Thrombocytopenia	12 (11.01%)		
Focal/spontaneous intestinal perforation (FIP/SIP)	9 (8.26%)		
Disseminated intravacular coagulation	2 (1.83%)		
Fetofetal transfusion	1 (0.92%)		

*P*-values are given for comparison between control group and corresponding affected group. Statistically significant values are shown in bold

**Table 2:** Statistical characteristics of regularly measured clinical parameters in entire study cohort

Clinical parameters			Study cohort				
Parameter	Units	Plausible range	Range	Mean ± Std	Q1	Q3	Median ± IQR
*Blood Gases and Acid-Base Status*							
pH	-	6.8–7.6	6.7–7.6	7.3 ± 0.1	7.2	7.4	7.3 ± 0.1
pCO_2_	mmHg	15–120	17.6–99.1	43.8 ± 11.0	36.2	49.5	42.1 ± 13.3
pO_2_	mmHg	20–150	19.9–144.7	53.6 ± 13.0	47.4	58.4	52.7 ± 11.0
SaO_2_	%	40–100	38.6–99.7	84.6 ± 11.3	79.3	92.9	87.8 ± 13.6
SpO_2_	%	40–100	39.7–100	94.9 ± 3.59	92	98	95 ± 6
HCO_3_^-^	mmol/L	5–40	7.7–38	20.1 ± 3.4	18	22	20.1 ± 4
BE	mmol/L	-25–20	-24.6–18.5	-6.1 ± 4.0	-8.3	-3.6	-5.9 ± 4.7
Lactat	mmol/L	0.2–15	0.2–14.11	2.1 ± 1.47	1.28	2.4	1.6 ± 1.1
*Circulatory parameters*							
MAP	mmHg	15–80	17–84	38.6 ± 8.74	33	43	38 ± 10
SYS	mmHg	20–110	22–100	49.8 ± 11.0	42	56	49 ± 14
DIA	mmHg	10–80	10–78	30.7 ± 8.4	25	35	30 ± 10
Heart rate	/min	80–220	97–220	161.4 ± 14.8	150	170	160 ± 20
Respiratory rate	/min	10–120	10–100	52.4 ± 11.1	44	60	52 ± 16
*Complete blood count*							
Hematocrit	%	20–70	19. 4–66	41.8 ± 6.6	37	45.2	41 ± 8.2
Hemoglobin	g/dL	8–22	8.6–22.4	14.1 ± 2.2	12.7	15.3	14 ± 2.6
Erythrocyte count	10^12^/L	2–7	1.9–6.1	4.0 ± 0.6	3.6	4.4	4 ± 0.8
Thrombocyte count	10^9^/L	20–900	17–840	179.7 ± 105.3	108	225	166 ± 117
*Inflammation*							
Leukocyte count	10^9^/L	0.5–80	0.6–73	13.4 ± 10.9	6.4	16.7	10.3 ± 10.3
Lymphocyte count	10^9^/L	0.5–80	0.44–81	19.0 ± 20.1	3	32	11 ± 29
CRP	mg/L	0–300	0.09–288.6	10.7 ± 29.4	0.4	6.8	1.1 ± 6.4
Temperatur	°C	30–42	30.7–38.8	37.1 ± 0.4	36.9	37.4	37.1 ± 0.5
*Coagulation*							
PTT	sec	20–185	32–181	83.6 ± 43.0	54.3	97.5	67 ± 43.3
INR	-	0.8–3	0.9–3.2	1.5 ± 0.4	1.2	1.6	1.3 ± 0.4
Quick	%	20–150	22–127	66.7 ± 22.7	49	80	65.5 ± 31
Fibrinogen	mg/dL	50–600	49–491	213.4 ± 112.6	133	272.3	192 ± 139.5
*Electrolytes and Chemistry*							
Blood glucose	mg/dL	20–700	25.7–678	123.2 ± 56.1	87	148	110 ± 61
Sodium	mmol/L	115–170	112–161	140.1 ± 5.8	136	143.7	140 ± 7.4
Potassium	mmol/L	2–9	2.2–9.1	4.4 ± 0.9	3.8	4.9	4.3 ± 1.1
Calcium	mmol/L	0.5–3.5	0.6–3.9	1.4 ± 0.4	1.2	1.4	1.3 ± 0.2
Bilirubin	µmol/L	0–500	0.19–59	5.3 ± 2.6	3.9	6.5	5.2 ± 2.6

In the control and all affected groups, maximum values of whole blood glucose levels (Table 3) at least once fell outside the safe limits of reported in the literature (Table 6). However, mean whole blood glucose levels in all affected groups were higher than in controls, Moreover, following IVH diagnosis, they became significantly higher than in the control group and even exceeded the whole blood hyperglycemia threshold of 125 mg/dL (Table 6).

**Table 3:** Gestational age, birth weight and whole blood glucose in control and affected groups

Group	Gestational age [weeks]			Birth body weight [g]			Whole blood glucose [mg/dL]		
	min	max	mean ± std (*p*-value)	min	max	mean ± std (*p*-value)	min	max	mean ± std (*p*-value)
Control (*n* = 43)	23 + 5	31 + 5	27.2 ± 2.2	500	1950	993 ± 314	28	378	108.41 ± 28.65
Affected									
IVH (I-IV) or cWMI (*n* = 66)	22 + 4	33 + 4	26.8 ± 2.8 (0.373)	490	1600	948 ± 325 (0.472)	26	678	***all days*** **121.02 ± 42.07** **(0.103)**
IVH (I-IV) (*n* = 56)	23 + 1	33 + 2	26.4 ± 2.4 (0.116)	490	1590	915 ± 309 (0.211)	26	678	***all days*** **124.98 ± 40.30** **(0.019)**
									*before* 115.71 ± 38.15 (0.372)
									***after*** **130.39 ± 47.03** **(0.007)**
cWMI (*n* = 25)	22 + 4	33 + 4	27.7 ± 3.2 (0.638)	520	1600	1012 ± 355 (0.779)	26	356	*all days* 116.68 ± 49.83 (0.869)
									*before* 113.44 ± 47.39 (0.945)
									*after* 115.32 ± 55.05 (0.955)

Statistically significant values are shown in bold

The assessment of the relationship between hyperglycemia and 32 regularly measured parameters revealed 21 parameters that were significantly (p-value < 0.05) associated with hyperglycemia (Table 4). Furthermore, elevated glucose levels showed a significant association with 8 medical diagnoses (Table 5), with mean values exceeding the hyperglycemia threshold of 125 mg/dL in three of these conditions (Table 6).

**Table 4:** Association of hyperglycemia (whole blood glucose level over 125 mg/dL) with different regularly measured clinical parameters

Parameter	no Hyperglycemia	Hyperglycemia	*p*-value
	mean ± std	mean ± std	
*Blood Gases and Acid-Base Status*			
pH	7.33 ± 0.05	7.26 ± 0.07	**< 0.001**
pCO_2_	40.68 ± 5.36	45.59 ± 6.42	**< 0.001**
pO_2_	53.97 ± 5.76	53.44 ± 5.97	0.445
SaO_2_	84.69 ± 7.79	85.77 ± 6.49	0.773
SpO_2_	95.78 ± 2.11	92.97 ± 2.05	**< 0.001**
HCO_3_^−^	20.86 ± 1.87	19.25 ± 2.12	**< 0.001**
BE	-4.65 ± 1.90	-7.23 ± 2.68	**< 0.001**
Lactat	2.01 ± 0.82	2.36 ± 1.07	**0.041**
*Circulatory parameters*			
MAP	41.19 ± 5.76	38.02 ± 4.56	**0.016**
SYS	53.87 ± 7.36	48.67 ± 5.73	**0.001**
DIA	33.07 ± 5.65	30.34 ± 4.14	**0.029**
Heart rate	160.52 ± 8.58	163.67 ± 10.74	**0.099**
Respiratory rate	52.51 ± 5.83	55.40 ± 8.91	**0.031**
*Complete blood count*			
Hematocrit	43.71 ± 5.01	41.27 ± 3.66	**0.034**
Hemoglobin	14.69 ± 1.59	13.98 ± 1.32	**0.061**
Erythrocyte count	4.06 ± 0.48	3.98 ± 0.31	0.659
Thrombocyte count	224.95 ± 81.99	167.47 ± 69.09	**< 0.001**
*Inflammation*			
Leukocyte count	12.33 ± 9.13	14.75 ± 7.95	**0.101**
Lymphocyte count	21.99 ± 20.54	14.67 ± 13.09	0.370
CRP	3.94 ± 5.96	14.07 ± 27.91	**0.016**
Temperature	37.12 ± 0.12	37.12 ± 0.08	0.907
*Coagulation*			
PTT	94.42 ± 56.21	75.37 ± 20.34	0.913
INR	1.39 ± 0.29	1.49 ± 0.37	0.465
Quick	65.13 ± 21.04	63.39 ± 17.59	0.782
Fibrinogen	255.69 ± 106.23	246.43 ± 106.47	0.561
*Electrolytes and Chemistry*			
Sodium	139.15 ± 2.85	141.23 ± 2.89	**< 0.001**
Potassium	4.60 ± 0.47	4.34 ± 0.38	**0.012**
Calcium	1.43 ± 0.28	1.41 ± 0.30	0.371
Bilirubin	5.82 ± 1.46	4.94 ± 1.09	**0.008**
*Cerebral circulation * *(numerically calculated)*			
CBF	14.61 ± 4.54	11.95 ± 3.19	**0.011**
CBF-CBF_mean_	-0.27 ± 4.14	0.36 ± 3.05	0.169
PtO_2_	16.26 ± 4.79	14.41 ± 3.37	**0.065**

Statistically significant (p-value < 0.05) and moderate (p-value < 0.1) values are shown in bold

**Table 5:** Association of whole blood glucose level with different medical diagnoses

Medical diagnosis	no diagnosis	with diagnosis	*p*-value
	mean ± std [mg/dL]	mean ± std [mg/dL]	
*Prenatal diagnosis*			
Chorioamnionitis/amniotic infection syndrome	111.47 ± 45.59	117.59 ± 33.51	**0.099**
Preterm premature rupture of membranes (PPROM)	115.36 ± 40.29	116.06 ± 35.40	0.683
In vitro fertilisation	113.25 ± 44.74	121.33 ± 47.08	**0.069**
Preeclampsia/EPH-gestosis	114.14 ± 35.83	126.94 ± 47.23	0.490
*Infant diagnosis*			
Respiratory distress syndrome (all grades)	93.95 ± 30.64	119.18 ± 37.38	**< 0.001**
Erythrocyte transfusion requirement	99.67 ± 22.60	126.83 ± 41.55	**< 0.001**
Sepsis	109.78 ± 35.78	128.42 ± 38.21	**0.011**
Bronchopulmonary dysplasia (BPD, all grades)	112.58 ± 39.48	124.68 ± 29.79	**0.022**
Respiratory Adaptation Disorder	112.66 ± 37.76	124.05 ± 35.87	**0.069**
Acidosis (respiratory, metabolic, or both)	114.23 ± 37.49	122.96 ± 37.32	0.236
Necrotizing enterocolitis (NEC)	115.44 ± 38.21	117.48 ± 33.89	0.639
Pulmonary hemorrhage	113.61 ± 34.89	135.66 ± 54.19	0.126
Thrombocytopenia	113.66 ± 37.45	140.66 ± 28.39	**0.014**
Focal/spontaneous intestinal perforation (FIP/SIP)	113.57 ± 32.87	145.76 ± 74.66	0.405

Statistically significant (p-value < 0.05) and moderate (p-value < 0.1) values are shown in bold

**Table 6:** Threshold values of hypo- and hyperglycemia

Reference	Hypoglycemia		Hyperglycemia		
	mg/dL	mmol/L	mg/dL		mmol/L
[17, 19]			plasma/serum: 150	plasma/serum: 8.3	
[19]			whole-blood: 125	whole-blood: 6.9	
[23]	whole-blood: 40	whole-blood: 2.2			
[18]	whole-blood: 46				
[19]	plasma/serum: 70				
[16]		plasma/serum: 2.6			

## Discussion

In order to identify a potential association of whole blood glucose level with the preterm brain injury, a cohort of 109 preterm infants with and without IVH/cWMI was analyzed and compared against hypo- and hyperglycemia thresholds from literature. Statistical analysis revealed that patients in all groups experienced episodes of hypo- and hyperglycemia. Furthermore, higher mean values in affected groups compared to controls, and mean values exceeding the hyperglycemia threshold of 125 mg/dL [19] following IVH, indicated that elevated whole-blood glucose levels is associated with the development of brain injury in preterm infants.

Results are in agreement with the observations of Wheeler et al. [24], who revealed higher glucose levels, though not significantly, in preterm infants with PVL. Therefore, it can be assumed that cWMI is associated with chronic metabolic dysregulation prior to brain damage. While cWMI is already associated with early postnatally altered glucose regulation, significantly elevated blood glucose levels in IVH tend to manifest only following the haemorrhage.

Hyperglycemia induces hyperosmolality within the vasculature, drawing fluid from the extravascular space into the blood vessels. The resulting volume and pressure fluctuations cannot be compensated by the fragile vessels of the germinal matrix, precipitating vessels rupture and subsequent IVH. Simovic et al. [25] demonstrated significantly higher rates of IVH in hyperglycemic infants (*p* = 0.007). A retrospective analysis of 255 very low birth weight preterm infants [14] revealed hyperglycemia in 44.7% of the entire cohort, with a significantly higher incidence of IVH compared to normoglycemic infants (*p* < 0.001). Following logistic regression also confirmed hyperglycemia as an independent risk factor for IVH (*p* < 0.05) [14].

Our analyses revealed an association between increased blood glucose levels with 8 medical diagnoses. Thus, significant or moderate associations were observed between hyperglycemia and following diagnoses: chorioamnionitis/amniotic infection syndrome, in vitro fertilisation, respiratory distress syndrome, erythrocyte transfusion requirement, sepsis, bronchopulmonary dysplasia, respiratory adaptation disorder, thrombocytopenia. This association was supported by corresponding statistically significant or moderate values in 21regularly measured parameters.

Our results demonstrated that hyperglycemia is significantly associated with CBF, its fluctuations CBF–CBF_mean_ and moderately associated with brain tissue partial pressure (PtO_2_). The connection to hyperglycemia lies in the fact that it is part of a systemic state of stress and instability that simultaneously affects circulation and cerebral perfusion. In preterm infants, cerebrovascular autoregulation is still immature, so CBF is highly dependent on systemic circulation. Therefore, even small hemodynamic changes, such as those observed here in conjunction with hyperglycemia, can lead to significant fluctuations in cerebral perfusion, thus increasing the risk of IVH and cWMI [26]. Although hyperglycemia is associated with a range of adverse outcomes in preterm infants, there is a lack of evidence for a causal relationship [27].

The relationship between acute hyperglycemia and CBF remains inconsistent across the literature. Nowaczewska et al. [28] demonstrated that acute hyperglycemia is associated with increased blood flow velocities, which normalize alongside blood glucose levels. By contrast, according to Powers et al. [29] a reduction in arterial glucose concentration below 2.0 mmol/L leads to an increase in CBF. Also, Duckrow et al. [30] reported a decrease in CBF during acute hyperglycemia in studies of adults, which they attributed to increased plasma osmolality. However, transient increases in CBF have also been documented in this context. These observations were made during hyperglycemia with changes to plasma osmolality. No direct osmolality measurements were taken, and glucose levels were within published ranges, so these mechanisms cannot be inferred, but they provide a potential physiological background for the associations. Although these mechanisms may offer insight into possible pathways, their direct applicability to preterm infants is limited due to developmental differences in cerebrovascular regulation.

Acidosis, present in approximately 21% of preterm infants in our cohort, was manifested as significantly elevated pCO₂, with decreased pH, HCO₃⁻, and a negative base excess. This overall indicates metabolic dysregulation with incomplete respiratory compensation. It could be both a marker of disease severity in the sense of a stress response and a potential contributing factor to brain injury. However, a clear causal relationship cannot be established. Marik et al. [31] describe stress hyperglycemia as a metabolic response to critical illness that reflects disease severity in adult patients.

Both acidosis and hyperglycemia are recognized risk factors for IVH and WMI [16, 32], therefore they may act synergistically to create a hemodynamically unstable, pro-inflammatory environment in which the immature germinal matrix vasculature and white matter are particularly susceptible to injury. Hyperglycemia can precipitate osmotic diuresis and electrolyte imbalances leading to metabolic acidosis [14], whilst metabolic acidosis has independently been identified as a risk factor for IVH in preterm infants [32–34].

Furthermore, hyperglycemia patients exhibit an increased inflammatory and stress response, demonstrated by significantly elevated CRP and leukocyte levels in our study. It is possible that stress hormones such as cortisol and adrenaline are released in greater quantities, thereby putting the metabolism into a state of stress. Glucose is released into the bloodstream in greater amounts and simultaneously absorbed less effectively into the cells, which can lead to hyperglycemia in adult patients [31]. However, it should be taken into account that the use of catecholamines is usually itself an expression of severe clinical instability and increased disease severity, as is often the case in preterm infants with IVH and WMI. In this context, the observed increased level of blood glucose can at least partially be interpreted as an expression of the underlying severity of the disease and the associated intensity of intensive care therapy.

Whilst no direct causal relationship exists between hyperglycemia and leukocyte, both may occur concurrently in preterm infants. Perinatal stress and infection elevate cortisol, which in turn raises blood glucose levels [35]. Immunological immaturity renders preterm infants particularly susceptible to infection, and hyperglycemia compounds this vulnerability by impairing leukocyte function, altering their counts, and promoting pro-inflammatory cytokine production [36, 37]. In the study by Turina et al. [37], these effects were described in adult patients, however their direct applicability to preterm infants is therefore limited.

Particularly noteworthy are the associations, although insignificant due to the small number of patients with this diagnosis in the cohort, of elevated glucose levels with FIP/SIP, which is itself linked to both PVL [38] and IVH [39]. Hyperglycemia disrupts the intestinal environment of preterm infants, promoting pathogenic bacterial colonization and precipitating severe inflammatory responses such as FIP/SIP. It is also frequently observed in infants with necrotizing enterocolitis (NEC) and is associated with increased long-term mortality and prolonged intensive care admission [40]. Corticosteroid therapy for FIP/SIP further elevates blood glucose, supporting the notion that hyperglycemia acts as a trigger rather than a consequence of these intestinal conditions [41]. At the same time, however, it must be taken into account that FIP/SIP and other severe systemic inflammatory conditions can themselves be associated with disrupted glucose homeostasis and secondary hyperglycemia. Further prenatal factors, such as chorioamnionitis may, induce inflammation and stress that provoke hyperglycemia and damage cerebral vasculature, potentially explaining the higher rates of IVH and cWMI observed in hyperglycemic infants in our cohort. Intrauterine stressors may additionally alter fetal glucocorticoid sensitivity [42].

Statistically significant changes in hematocrit, hemoglobin, sodium, potassium and bilirubin create a coherent picture: inflammation, metabolism, electrolyte balance, and coagulation may all be affected. This interplay is described in the literature by McCowen et al. [43] They describe that stress-induced hyperglycemia frequently occurs in critically ill adult patients and results from an increased release of stress hormones and inflammatory substances (e.g., TNF-α, interleukin-1). This leads to increased glucose production in the liver and reduced insulin uptake of glucose into body cells. Kumar et al. [44] and Li et al. [45] further reported that metabolic disturbances associated with diabetes disrupt coagulation homeostasis, promoting thrombus formation, thrombocyte hypersensitivity, and impaired fibrinolysis in adult patients. Hyperglycemia is thus more likely a component of this systemic stress response than an isolated event.

## Limitations of the study and future work

Interpretation of the current findings remains difficult because severe prematurity itself is closely associated with systemic instability, sepsis, intensive respiratory support, circulatory impairment, and treatment-related hyperglycemia. In daily NICU practice, these factors are often deeply intertwined. Therefore, it remains challenging to distinguish whether hyperglycemia represents an independent contributor to brain injury or rather a marker of overall physiological instability.

With regard to our statistical analysis, several limitations should be mentioned. Thus, although IVH and cWMI are often categorised as types of early-onset brain injury, they may result from different pathophysiological mechanisms and vary in terms of timing of diagnosis. Also, this study did not investigate separately mild (grade I–II) and severe (grade III–IV) IVH. Additionally, our cohort did not include the diffuse form of WMI, diagnosed only with cerebral MRI. These differences in diagnoses and their severity should be considered when interpreting parameters and observed associations discussed herein.

In addition, our study cohort was composed of patients with a high incidence of IVH/cWMI, keeping in mind the objectives of our study, which were to investigate the association between whole-blood glucose levels and preterm brain injury. However, this does not reflect the actual prevalence in an unselected population of preterm infants.

Furthermore, since glucose levels were measured within a defined, early postnatal time window, the influence of comorbidities occurring later during the hospital stay on the analysis is limited. As previous studies have suggested, nutritional management, glucose infusion strategies, and medical interventions can have a significant impact on early glucose levels in extremely preterm infants [1, 9]. These treatment-related factors highlight the need for future studies to distinguish more clearly between therapy-related and disease-related mechanisms of early hyperglycaemia.

In our analysis, glucose measurements were aggregated to a daily mean value for each infant, rather than treating each individual measurement as a separate data point. This approach was adopted to avoid pseudoreplication, assuming that repeated measurements within the same infant on the same day are not statistically independent. However, it should be taken into account that the use of daily averages may limit the representation of short-term glucose extreme values compared to an analysis of individual measurements. Thus, the REACT study by Beardsall et al. [46] showed that continuous glucose monitoring captures a significant number of out-of-range glucose values that might have gone undetected with intermittent measurements alone. These results suggest that pronounced diurnal fluctuations are a characteristic feature of glucose regulation in extremely and very preterm infants.

Also, as the present study aimed to investigate the association between early whole blood glucose disturbances and IVH/cWMI. the MAGE, an established measure of glycemic variability, was not applied because focus was on the association between glucose levels and IVH/cWMI, not temporal analysis.

Our analyses revealed that statistically significant increased blood glucose levels were observed after ultrasound diagnosis in the IVH group and before ultrasound diagnosis in the cWMI group. However, timing of the ultrasound diagnosis does not necessarily correspond to the actual onset of brain injury. This is particularly relevant for cWMI, as cystic changes require time to become detectable on cranial ultrasound. Therefore, glucose values described as occurring ‘before’ or ‘after’ diagnosis should strictly refer to the period before or after the ultrasound diagnosis, rather than the onset of injury.

Although the type and amount of glucose intake, as well as insulin therapy, are clinically relevant factors, these were outside the primary scope of this study and were not analysed separately. The primary aim of the study was not to evaluate therapeutic strategies for glucose control or varying glucose administration regimens, but rather to investigate elevated blood glucose levels as a potential marker of neurological vulnerability in very preterm infants. In light of these considerations, future studies should employ multivariate logistic regression or machine learning methods to adjust for key clinical confounders, based on the parameters identified in the present study as significantly associated with IVH and WMI.

## Conclusion

This study compared whole blood glucose levels in preterm infants with and without brain injury (IVH and cWMI). While episodes of hypo- and hyperglycaemia were observed across all groups, mean values of whole blood glucose levels in preterm infants diagnosed with IVH/cWMI were higher than in controls indicating that elevated glucose levels is associated with preterm brain injury. Mean values were higher both before and after diagnosis of IVH or cWMI, and even exceeded the hyperglycemia threshold of 125 mg/dL, after IVH diagnosis. Furthermore, a significant or moderate association was found between elevated blood glucose level and 8 medical diagnoses, as well as 21 routinely measured parameters. These findings emphasize that glucose levels often fluctuate in clinically relevant situations and therefore require careful monitoring as part of routine neonatal care.

## Data Availability

The data analysed in this study is subject to the following licenses/restrictions: raw data supporting the conclusions of this manuscript will be made available to any qualified researcher upon request to the corresponding author. Requests to access these datasets should be directed to renee.lampe@tum.de.

## Code availability

All calculations presented in the paper were produced with the standard software package MATLAB2025b.

## Abbreviations

CBF: Cerebral blood flow
Cwmi: Cystic white matter injury
IUGR: Intrauterine growth restriction
FIP/SIP: Focal/spontaneous intestinal perforation
IVH: Intraventricular hemorrhage
MAGE: Mean amplitude of glycemic excursions
MAP: Mean arterial pressure
NEC: Necrotizing enterocolitis
PPROM: Preterm premature rupture of membranes
PtO_2_: Partial pressure in brain tissue
SYS: Systolic blood pressure
WG: Weeks gestation
